# High Sensitivity Microfiber Interferometer Sensor in Aqueous Solution

**DOI:** 10.3390/s20174713

**Published:** 2020-08-21

**Authors:** Saad Hayatu Girei, Hong Ngee Lim, Muhammad Zamharir Ahmad, Mohd Adzir Mahdi, Ahmad Rifqi Md Zain, Mohd Hanif Yaacob

**Affiliations:** 1 Wireless and Photonics Networks Research Centre, University Putra Malaysia, Serdang 43400, Selangor, Malaysia; gireisaad3@gmail.com (S.H.G.); mam@upm.edu.my (M.A.M.); 2 Department of Chemistry, Faculty of Science, University Putra Malaysia, Serdang 43400, Selangor, Malaysia; hongngee@upm.edu.my; 3 Biotechnology and Nanotechnology Research Centre, MARDI Headquarters, Serdang 43400, Selangor, Malaysia; zamharir@mardi.gov.my; 4 Institute of Microengineering and Nanoelectronics (IMEN), Universiti Kebangsaan Malaysia (UKM), Bangi 43650, Selangor, Malaysia; rifqi@ukm.edu.my

**Keywords:** ammonia-nitrogen, microfiber interferometer, optical-fiber sensor

## Abstract

The need for environmental protection and water pollution control has led to the development of different sensors for determining many kinds of pollutants in water. Ammonia nitrogen presence is an important indicator of water quality in environmental monitoring applications. In this paper, a high sensitivity sensor for monitoring ammonia nitrogen concentration in water using a tapered microfiber interferometer (MFI) as a sensor platform and a broad supercontinuum laser as the light source is realized. The MFI is fabricated to the waist diameter of 8 µm producing a strong interference pattern due to the coupling of the fundamental mode with the cladding mode. The MFI sensor is investigated for a low concentration of ammonia nitrogen in water in the wide wavelength range from 1500–1800 nm with a high-power signal provided by the supercontinuum source. The broad source allows optical sensing characteristics of the MFI to be evaluated at four different wavelengths (1505, 1605, 1705, and 1785 nm) upon exposure towards various ammonia nitrogen concentrations. The highest sensitivity of 0.099 nm/ppm that indicates the wavelength shift is observed at 1785 nm operating wavelength. The response is linear in the ammonia nitrogen range of 5–30 ppm with the best measurement resolution calculated to be 0.5 ppm. The low concentration ammonia nitrogen detected by the MFI in the unique infrared region reveals the potential application of this optical fiber-based sensor for rivers and drinking water monitoring.

## 1. Introduction

Water is a widely used solvent in chemical and biological processes. Researchers have used optical fiber sensors and water as the solvent for sensing various chemicals such as ethanol [1], isopropanol [2], nitrates [3], and ammonia [4] in aqueous solutions. The sensing is based on a change of the refractive index (RI) of the aqueous solution. Microcomponents such as ammonia nitrogen in water provide important information about water quality in rivers and water supply processing plants as well as in drinking water [5]. High concentration of ammonia nitrogen in water can increase eutrophication which contributes to adverse effects for humans and aquatic life [6]. Moreover, concentrations ranging as low as 0.2–2.0 ppm can be lethal to fish species [7]. Major industries such as the pharmaceutical and chemical industries, fertilizer production, sewage treatment plants, and cattle excrement are some of the sources of ammonia nitrogen released into the environment [8]. In this regard, control of ammonia nitrogen effluent discharge limits to the environment is critical. Stipulated effluent discharge limit for ammonia nitrogen varies from one country to another. For example, in Malaysia, the acceptable discharge limits for ammonia nitrogen are 5 and 10 ppm for enclosed water bodies and rivers, respectively [9]. Therefore, a high sensitivity sensor is required for detecting a low concentration of ammonia nitrogen in water for environmental, health, and industrial safety.

Microfiber interferometer (MFI) sensors have been drawing the attention of researchers in the scientific community due to their outstanding properties such as excellent geometry, material uniformity, high index contrast between the optical fiber and the surrounding environment, strong evanescent field, and high sensitivity [10]. These features make them an ideal candidate for detecting slight changes in RI of an aqueous solution. In recent years, various configurations of MFI-based sensors have been used in determining RI changes in various aqueous solutions. These configurations include thin-core fiber (TCF) interferometer [11], grating-based MFI [3], single-mode multimode single-mode (SMS) MFI [12], photonic crystal fiber-based interferometer [13], and as-drawn taper-based interferometers [14]. Zhao et al. designed a SMS fiber structure for RI sensing with a measured sensitivity of 286.2 nm/RIU. Zhoa et al. [13] designed a tapered photonic crystal fiber (PCF)-based interferometer for RI measurement with a sensitivity of 281.6 nm/RIU. In 2017, Pham et al. [15] used etched fiber Bragg grating (e-FBG) to detect nitrate concentration in water with a limit of detection (LOD) of 3.0 ppm. Recently, Huang et al. [16] presented MFI realized by splicing a section of TCF between two SMS coated with a PAH/PAA multilayer film for ammonia nitrogen monitoring in water with a sensitivity of 0.031 nm/ppm. However, the TCF and SMS-based devices involve a complicated fabrication technique and PCFs are expensive. The as-drawn tapered MFI structure is simpler, straightforward, and cost-effective compared to grating-based MFI, TCF, PCF, and SMS based devices. 

In this paper, an ultrasensitive MFI sensor based on a standard single-mode optical fiber (SMF) for monitoring low concentration of ammonia nitrogen (5–50 ppm) in water is experimentally demonstrated. The sensor is investigated in a wide wavelength range of 1500–1800 nm. The results presented here indicate that the MFI sensor can detect a low concentration of ammonia nitrogen in water with a 0.5 ppm limit of detection.

## 2. Fabrication and Principle

**Figure 1:**
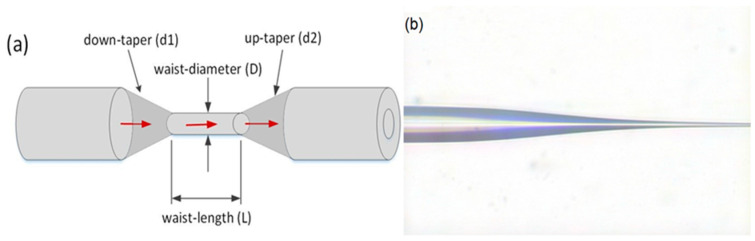
(**a**) Schematic of microfiber interferometer (MFI); (**b**) microscopic image of MFI.

The MFI is fabricated by heating and pulling SMF (SMF-28, Lucent tech., NJ, USA) using a Vytran^TM^ glass-processing machine (GPX-3400, Morganville, NJ, USA). First, a section of the SMF fiber is fixed on two translational stages by a fiber holder. While the center of the SMF is heated by a filament, the two translational stages simultaneously move in the opposite direction to stretch the fiber down from the initial diameter to the final diameter. Figure 1a shows the schematic structure of the proposed MFI fabricated from standard SMF. The MFI consists of down-taper section d1, waist-length L, waist-diameter D, and up-taper section d2. Figure 1b shows the microscopic image of the down-taper, and part of the waist length of the MFI. Tapered SMF can be either adiabatic or non-adiabatic depending on the taper angle. An adiabatic taper has a very small local change in the taper radius (small taper angle) leading to the main portion of the power remaining in the fundamental mode, and therefore it does not couple to higher-order modes as it propagates through the fiber. On the other hand, the non-adiabatic taper has an abrupt taper angle and is capable of transferring energy from the fundamental mode propagating through the normal fiber to the cladding modes in the down-taper region [17]. As can be seen in Figure 1b, the fabricated taper is non-adiabatic because of its sharper taper angle. This coupled the fundamental mode to cladding mode thus creating a strong interference pattern that behaves as a modal interferometer. 

When light propagates through the down-taper and due to its relatively sharp taper angle, the fundamental mode will be coupled to the high-order cladding mode. Hence, more than one mode will be propagating along the taper waist. In the up-taper region, parts of the cladding modes are coupled back to the core, resulting in interference patterns. Output light intensity I of the transmission is given by [18,19]:

(1) $$I=I_{1}+I_{2}+2\sqrt{I_{1}I_{2}}\;\cos\left(\Delta \emptyset \right)$$

where I_1_ and I_2_ are the light intensity of the fundamental mode and cladding mode, respectively. Δ∅ is the phase difference between the interfering modes and is expressed as:

(2) $$\Delta \emptyset =\frac{2\pi \Delta n_{eff}L}{\lambda }$$

where λ is the central wavelength of the light source, L is the waist length of the tapered region, and $\Delta n_{eff}$ is the effective refractive index difference between the fundamental and cladding mode $\left(\Delta n_{eff}=n_{co}-n_{cl}\right)$. When Δ∅ = 2mπ, an interference maximum could be observed at the m-order peak wavelength given by:

(3) $$\lambda_{m}=\frac{\Delta n_{eff}L}{m}$$

A change in the behavior of the surrounding environment will influence the effective refractive indices between the fundamental and cladding mode, $n_{eff}$ will correspondingly change to $\left(\Delta n_{eff}+\delta \left(\Delta n_{eff}\right)\right)$. Consequently, peak wavelength shifts δλ in the position of the m-order peak could be observed in the interference spectrum. The peak wavelength shifts and the associated phase shift δ∅ can be expressed as follows respectively:

(4) $$\delta \lambda =\delta \left(\frac{\Delta n_{eff}L}{m}\right)=\left(\frac{\delta L}{L}+\frac{\delta \left(\Delta n_{eff}\right)}{\Delta n_{eff}}\right)\lambda$$

(5) $$\delta \emptyset =\frac{2\pi \left(\Delta n_{eff}\delta L\left(\Delta n_{eff}\right)\right)}{\lambda }$$

## 3. Experiment and Results

**Figure 2:**
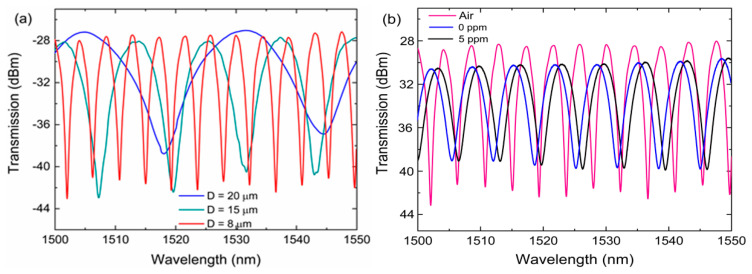
Transmission spectra of (**a**) MFI with different waist diameter D (**b**) 8 µm MFI in air, water, and 5 ppm ammonia nitrogen.

Figure 2a shows the transmission spectra of MFI in air in the wavelength range of 1500–1550 nm with different waist diameter (D), waist-length (L) = 15 mm, up-taper = down-taper = 3 mm. The spectra were obtained by optical spectrum analyzer (OSA, AQ6375, Yokogawa, Tokyo, Japan) and broadband supercontinuum light source. As can be seen from Figure 2a, the trend of the spectrum shows that the free spectral range (FSR) decreases as the waist diameter decreases. The MFI with waist-diameter 20, 15, 8 µm has a FSR of 26.60, 12.38, and 4.86 nm, respectively. The MFI with D = 8 µm exhibited close fringes and has narrow dips with the highest extinction ratio of around 15.12 dB. These features are important for sensing application because they help to improve the accuracy and resolution of any sensor [20]. Figure 2b shows the spectrum of the 8 µm MFI in air and immersed in 5 ppm ammonia nitrogen. Comparing the spectrum in air and 5 ppm ammonia nitrogen, the intensity power loss after immersion is estimated to be ~2.0 dB with extinction ration reduced to around 10.0 dB which is suitable for sensing. The FSR of the spectrum after immersion in solution has increased from 4.86 nm to 6.58 nm, with reference to Equation (4), this occurrence is reasoned with the reduction of $\Delta n_{eff}$ when $n_{cl}$ increases. The spectrum still exhibited narrow dips, which are critical for monitoring wavelength shift changes with high accuracy. For this reason, the MFI with D = 8 µm was chosen for this experiment. The interference pattern of the spectrum is due to mode coupling between the fundamental mode and cladding modes. 

**Figure 3:**
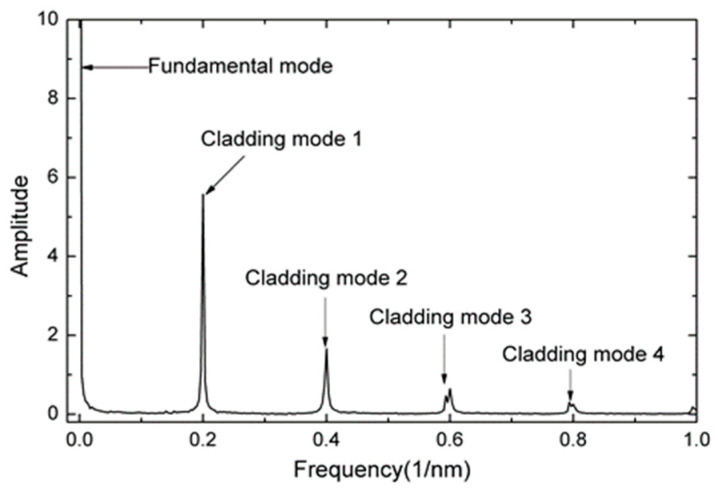
Spatial frequency spectrum of the MFI.

To determine information on mode distribution and its effects on the interferometric spectrum, Fast Fourier Transform (FFT) formula is used on the measured spectrum to obtain the spatial frequency spectrum of the MFI, as shown in Figure 3. It can be observed that, apart from the fundamental mode, dominantly excited cladding mode (cladding mode 1) and weakly excited cladding mode (cladding mode 2, 3, and 4) were also stimulated in the spatial frequency spectrum. It is believed that interference occurs mainly between the fundamental mode and dominantly excited mode (cladding mode 1). The other cladding mode also contributes to the interference but is negligible due to relatively low light intensity propagates in the cladding. Thus, it can be concluded that the interference pattern is due to the combined action of the fundamental mode and cladding mode 1.

**Figure 4:**
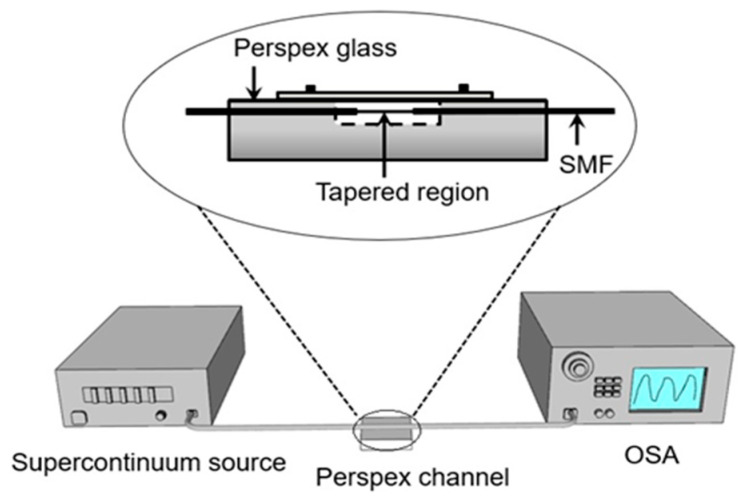
Experimental set-up for ammonia nitrogen measurement.

Figure 4 shows the schematic diagram of the experimental setup used for the ammonia nitrogen measurement. A high-power supercontinuum source (SC-5, YSL Photonics) covering a broad spectrum range (470–2400 nm) is used to launch light into the tapered MFI. The other end of the MFI was connected to OSA (AQ6375, Yokogawa, Tokyo, Japan). Ammonia nitrogen solution with different concentrations was obtained by diluting ammonia nitrogen standard solution (NH3-N) with pure water. The MFI was fixed and tautened on a narrow channel made from Perspex® glass. The narrow channel had a volume of 1000 µl where the MFI as a whole was immersed into the ammonia nitrogen solution at a concentration from 5–50 ppm with 5 ppm increment. The ammonia nitrogen solution is pumped slowly into the narrow channel through an inlet opening by a micro-injection pump. The solution is drained out through an outlet opening by an automated syringe pump. In each concentration, the measurement of the spectral shift from OSA was recorded after the MFI sensor was submerged in the solution for 5 min.

**Figure 5:**
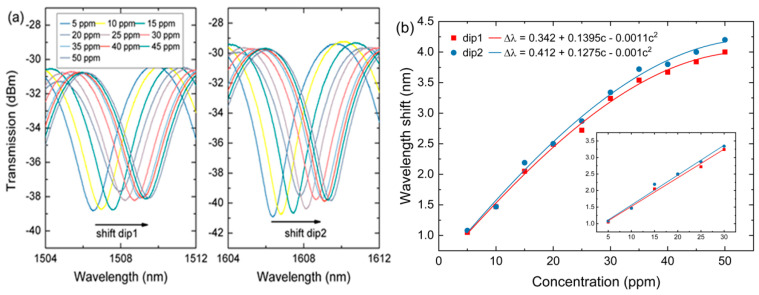
(**a**) Transmission spectra of MFI with different ammonia nitrogen concentration for dip1and dip2. (**b**) The linear relation between dip position and concentration for dip1 and dip2, the inset is the linear fit for low concentrations of 5–30 ppm.

The transmission spectra of the MFI sensor that monitored around 1505 nm (dip1) and around 1605 nm (dip2) is shown in Figure 5a. As can be seen, the dips in dip1 and dip2 shift toward longer wavelengths as ammonia nitrogen concentration increases from 5–50 ppm. For example, a concentration of 10 ppm produces a wavelength shift of 0.42 nm and 0.39 nm to the right at dip1 and dip2, respectively. The RI of the core, $n_{co}$, does not change and therefore continuous increase in the refractive index of the cladding, $n_{cl}$, will lead to an increase in $\delta \left(\Delta n_{eff}\right)$. Consequently, according to Equation (3), a larger δλ is obtained which explains the redshift observation. Figure 5b shows a nonlinear relation between dip shift (dip1 and dip2) and ammonia nitrogen concentration. The sensitivity of the sensor decreases with the increase of ammonia nitrogen concentration. The saturation of the sensitivity might be attributed to the limited tapered fiber sites for the interaction, particularly at higher ammonia nitrogen concentrations. As a result, an increase of ammonia nitrogen concentrations produces lesser optical response magnitude [20]. Their respective sensitivities obtained at dip1 and dip2 are 0.0666 nm/ppm and 0.0703 nm/ppm. The nonlinearity curve of the transmission wavelength versus ammonia nitrogen concentration at dip1 is fitted as:

(6) $$\Delta \lambda =0.342+0.1395c-0.0011c^{2}$$

For dip2, the nonlinearity curve is fitted as:

(7) $$\Delta \lambda =0.412+0.1275c-0.001c^{2}$$

where Δλ is the change in wavelength shift and *c* is the ammonia nitrogen concentration. As can be observed, the sensor starts to reach saturation at a concentration of 35 ppm. The inset in Figure 5b shows the linear fit plot for low concentrations from 5–30 ppm for dip1 and dip2. 

**Figure 6:**
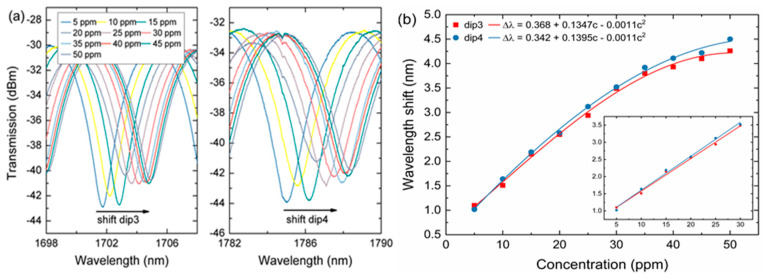
(**a**) Transmission spectra of MFI with different ammonia nitrogen concentration for dip3 and dip4. (**b**) The linear relation between dip position and concentration for dip3 and dip4, the inset is the linear fit for low concentrations of 5–30 ppm.

Figure 6a shows the transmission spectra of the interference dips around 1705 nm (dip3) and 1785 nm (dip4) of the MFI sensor. Similarly, it is easy to see the transmission spectra have undergone a redshift. Figure 6b shows the measured data and plot for the nonlinear fitting for dip3 and dip4 indicating the sensitivities of 0.0724 nm/ppm and 0.0768 nm/ppm. The nonlinearity curve of the transmission wavelength versus ammonia nitrogen concentration at dip3 is fitted as:

(8) $$\Delta \lambda =0.368+0.1347c-0.0011c^{2}$$

For dip4, the nonlinearity curve is fitted as:

(9) $$\Delta \lambda =0.342+0.1395c-0.0011c^{2}$$

where Δλ is the change in wavelength shift, and *c* is the ammonia nitrogen concentration. The inset in Figure 6b shows the response of the proposed MFI in the low concentration range of 5–30 ppm. At the interference dip3, the sensitivity obtained is 0.0951 nm/ppm, for which the linear fit is 0.995. At interference dip4, the sensitivity obtained is 0.099 nm/ppm, for which the linear fit is 0.994. The high sensitivity of the MFI sensor is due to the excitation of higher-order cladding mode, which is the key in the formation of a strong interference pattern. Compared with the other dips (dip1, dip2, dip3), dip4 exhibits a slightly higher dip shift under the same amount of changes in ammonia nitrogen concentration in water. This shows that the interference peaks have different sensitivities on the same RI changes [19]. This might have resulted from the behavior of several cladding modes whose effective index might have a different dependency on any environmental changes.

**Figure 7:**
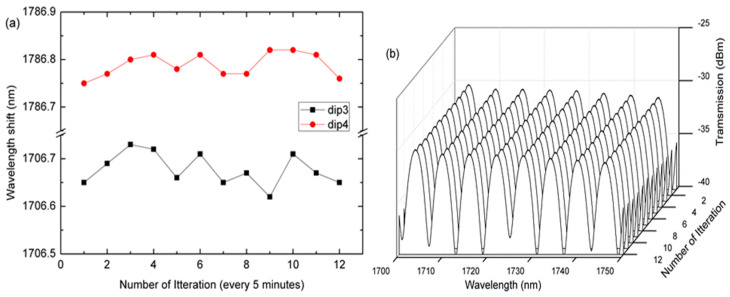
(**a**) Stability of the MFI at dip3 and dip4 immersed in 20 ppm ammonia nitrogen for 1 h. (**b**) The stability of the MFI transmission spectrum for 1 h immersed in 20 ppm ammonia-nitrogen.

We also evaluated the stability of the sensor by immersing it into ammonia nitrogen for one hour. Figure 7a shows the stability of the MFI sensor at 20 ppm for dip 3 and dip4. The standard deviation of the wavelength transmission spectrum at dip3 and dip4 was 0.03 nm and 0.02 nm, respectively. Figure 7b shows the stability of the MFI transmission spectrum for the one-hour period during which the ammonia nitrogen remained relatively unchanged at 20 ppm.

## 4. Discussion

The results in this work indicate that this sensor can be used as a highly sensitive measurement for low concentrations of ammonia-nitrogen. The range 5–30 ppm can be of interest in measuring ammonia nitrogen levels in effluent discharge for environmental monitoring. Regarding the OSA resolutions of 0.05 nm in our experiment, the minimum detectable concentration changes for the MFI sensor obtained was 0.5 ppm. The LOD of the current sensor was below the maximum ammonia nitrogen level allowed for a closed water body by the Malaysia Department of Environment (DOE) [9]. This means by using OSA with a high resolution, this method is appropriate for monitoring ammonia nitrogen concentrations in drinking water as well as in wastewater. The LOD of the current MFI sensor with a resolution of 0.05 nm is lower than previously related sensors reported in [3] with a resolution of 0.01 nm. Moreover, it has clear sensitivity advantages to other related sensors reported in the literature. For measurements of various forms of nitrogen in water, the comparison is shown in Table 1.

## 5. Conclusions

In summary, a tapered MFI sensor based on RI change of aqueous solution with high sensitivity for ammonia nitrogen monitoring in water was demonstrated at a room temperature operation. The sensor was realized by tapering standard SMF, producing an interferometric device with an extinction ratio of up to 15.12 dB. The sensor was investigated in a wide wavelength range from 1500–1800 nm by monitoring the shift of four interference dips. By using a wavelength resolution of 0.05 nm, a detection limit of 0.5 ppm was obtained. These findings are important in investigating the selectivity of the proposed sensor towards ammonia nitrogen in water by integrating ammonia sensitive materials to the taper waist region of the MFI. The characteristics of high sensitivity, easy fabrication, and robustness make this MFI sensor a potential candidate for detecting ammonia nitrogen in liquid surrounding such as in drinking water and seawater

## Figures and Tables

**Table 1:** Comparison between fabricated MFI sensor and other related sensors.

Sensor	Analyte	LOD (ppm)	Sensitivity (nm/ppm)	Ref
Tapered SMF	Sodium nitrate	1.34	0.014	[14]
Etched FBG	Nitrate	3.0	0.0035	[3]
LPG	Ammonia	0.1	-	[21]
Etched FBG	Ammonia	-	0.171	[22]
Tapered SMF	Ammonia-nitrogen	0.5	0.0768	This work
